# Forest Health Status in Europe

**DOI:** 10.1100/tsw.2007.17

**Published:** 2007-03-21

**Authors:** Martin Lorenz, Volker Mues

**Affiliations:** Federal Research Centre for Forestry and Forest Products, Leuschnerstr. 91, D-21031 Hamburg, Germany

**Keywords:** Europe, forest health, defoliation, deposition, sulfur

## Abstract

Forest health status in Europe is assessed by the International Cooperative Programme on Assessment and Monitoring of Air Pollution Effects on Forests (ICP Forests). Established by the Convention on Long-Range Transboundary Air Pollution (CLRTAP) under the United Nations Economic Commission for Europe (UNECE), the ICP Forests has been monitoring forest condition in close cooperation with the European Commission (EC) for 20 years. The present paper describes the latest results of the deposition measurements on permanent monitoring plots and of the extensive defoliation sample survey. The findings reveal marked spatial patterns in bulk and throughfall depositions of nitrate (N-NO_3_), ammonium (N-NH_4_), and sulfate (S-SO_4_), as well as an obvious decrease in bulk and throughfall deposition of sulfate. Latest analyses of defoliation data confirm previous results, indicating a high correlation with weather extremes.

### INTRODUCTION

Forest health in Europe has been monitored by means of harmonized methods since 1986 as a response to the growing concern that defoliation observed in many forested areas could be caused by air pollution[1,2]. Monitoring is coordinated by the International Cooperative Programme on Assessment and Monitoring of Air Pollution Effects on Forests (ICP Forests) of the Convention on Long-Range Transboundary Air Pollution (CLRTAP) under the United Nations Economic Commission for Europe (UNECE) in close cooperation with the European Union (EU). Today 41 countries are participating, including the U.S. and Canada, thus rendering one of the largest monitoring programs of its kind worldwide. All participating countries are represented in the Task Force of ICP Forests, which is chaired by Germany. Germany hosts the Programme Coordinating Centre (PCC) at the Federal Research Centre for Forestry and Forest Products (BFH) in Hamburg, which coordinates the monitoring progam, the database management, and substantial parts of the evaluation and reporting. ICP Forests aims to provide scientific knowledge on the effects of air pollution on forests as a basis for air pollution control policies under CLRTAP. For this purpose, ICP Forests pursues the following two objectives:

1. Assessments of the spatial and temporal variation in forest condition on the European level and the corresponding relationships to environmental factors, air pollution in particular.

2. Contribution to a better understanding of forest ecosystem response to environmental factors with a focus on air pollution.

Results of ICP Forests indicate effects of ozone on leaves of forest trees, shrubs, and herbs[3], as well as impacts of sulfur, nitrogen, and heavy metals on forest soils[3,4,5,6]. Ozone-induced injury was found on the foliage of several tree species and numerous ground vegetation species, especially in the Mediterranean countries[3]. Defoliation was found to be explainable mainly by natural factors, such as tree age, weather extremes, as well as insects and fungi, whereas correlations between defoliation and depositions of air pollutants were weak[7]. However, recent studies reveal increasing defoliation at sites at which critical loads for acidity and nitrogen are exceeded. The calculation of critical loads and their exceedances constitutes one of the driving forces of air pollution abatement policies by CLRTAP under UNECE. This approach requires measurements of atmospheric deposition. Measurements of depositions are also required for reviewing the effectiveness of CLRTAP policies, and for the calibration and validation of models of large-scale deposition. The present paper describes the latest results of the extensive defoliation survey and of the deposition measurements by the ICP Forests.

### METHODS

The two objectives of ICP Forests are met by means of two different monitoring approaches. A large-scale monitoring approach aims to assess the spatial and temporal variation of forest condition across Europe. It is pursued on more than 6,100 permanent sample plots allocated as far as possible on a systematic 16- × 16-km sample grid (Level I). On all Level I plots, annual crown condition assessments are carried out. On 5,289 plots, soil surveys were conducted mainly in the years 1993–1995. Moreover, foliage chemistry surveys were conducted on 1,497 plots, mainly in the years 1992–1997. The systematic sampling allows for a representative description of the current state and changes in the time and space domain of forest condition. For gaining insight into ecosystem response to environmental factors, the sampling approach was supplemented by an intensive monitoring approach. More than 860 intensive monitoring plots (Level II) were selected in the most important forest ecosystems of the participating countries. On those Level II plots, the assessment of a comprehensive set of monitoring parameters was implemented that facilitates a detailed and sensitive study of cause-effect relationships. The intensive monitoring aims at crown condition, soil condition, soil solution chemistry, foliar chemistry, tree growth, tree phenology, ground vegetation, meteorological condition, ambient air quality, and deposition. Not all of the respective monitoring activities are conducted on the entire set of Level II plots. All assessments on Level I and Level II plots within the program are based on harmonized methods documented in a regularly updated manual[8].

The spatial and temporal variation of defoliation was derived from the Level I monitoring network. The analysis of the temporal variation had to take into account variations due to the progressing inclusion of newly participating countries, because forest condition is subject to pronounced differences between countries and respective ecoregions. For this reason, the development of defoliation was calculated for a subset of those countries having started their defoliation assessments at the latest in 1990, covering a time series of 16 years (1990–2005). These countries were Belgium, Denmark, Germany (west), Hungary, Ireland, Latvia, Poland, Portugal, Slovak Republic, Spain, Switzerland, and The Netherlands.

At Level II, bulk deposition is measured in the open field in the neighborhood of the plots. Total deposition under canopy is derived from throughfall measurements with stemflow added where necessary (e.g., for common beech because of its smooth bark). Canopy exchange (uptake and leaching) can be taken into account by means of suitable models[4].

For the present study, annual bulk and throughfall depositions of nitrate (N-$NO_3^-$), ammonium (N-$NH_4^+$), sulfate (S-$SO_4^{2-}$), $Na^+$, $Cl^-$, and $Ca^{2+}$ were calculated as the arithmetic mean over the years 1999–2001 for each Level II plot in kg • $ha^{-1}$ • $year^{-1}$. Changes over time were calculated over the 1998–2003

period. In the light of data availability, the choice of this period permitted the inclusion of a maximum number of plots. Only those plots were involved in the study on which deposition had been measured continuously over that period, with 1 month maximum of measurements missing. Data of missing days were replaced by the average daily deposition of the respective year. With the years of assessment as predictor and annual deposition as target variable, linear relationships were obtained for each plot. Conclusions about temporal trends in ion depositions based on such a short period must be considered with care. Depending on data availability, between 202 and 267 Level II plots were included in the analysis. Canopy exchange was not considered.

## RESULTS

### Crown Condition

Several of the main tree species show an increase in defoliation from 1990–2005 (Fig. 1). These species are *Pinus pinaster* (increase from 13.2–18.9% mean defoliation), *Fagus sylvatica* (17.9–22.2%), *Quercus ilex* and *Q. rotundifolia* (13.8–23.8%), as well as *Q. robur* and *Q. petraea* (21.0–25.5%). Defoliation of *Picea abies* varied around 23% without any clear trend. Previous multivariate and geostatistical studies[7] reveal significant correlations between defoliation and tree age, biotic factors, and weather extremes. Weather extremes are also the main factor influencing defoliation in recent years. Due to the severe heat and drought in the summer of 2003, crown condition of all main species except *Pinus sylvestris*, *Q. ilex*, and *Q. rotundifolia* deteriorated rapidly from 2003–2004 in southern Finland, southernmost Sweden, central and southern Germany, some parts of France, and the entirety of Bulgaria. From 2004–2005, *F. sylvatica*, *Picea abies,* as well as *Q. robur* and *Q. petraea* showed recuperation.

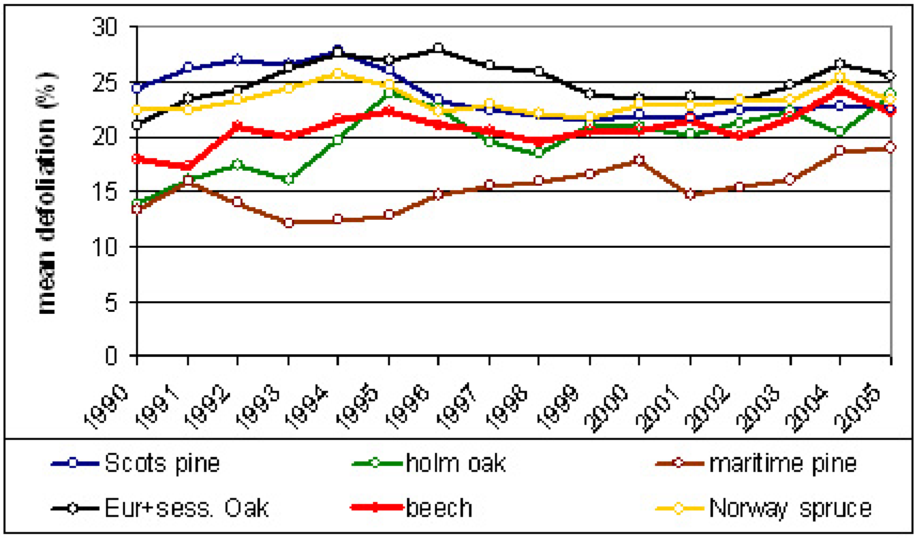

**FIGURE 1.** Development of mean defoliation of the six most common species.

Only for *Pinus sylvestris* was a significant relationship between defoliation and sulfur deposition found[9]. In Fig. 1, the high defoliation of *P. sylvestris* (27.7%) reflects the high share of sample trees in areas of previously high sulfur depositions, particularly in parts of Poland, the Czech Republic, the Slovak Republic, Germany, and the Baltic States. From 1994–2005, defoliation of *P. sylvestris* decreased from 27.7–22.6%. This clear recovery renders *P. sylvestris* the only species among the main species showing better crown condition today than in the early 1990s. In their national reports to ICP Forests, some of the above-mentioned countries have attributed the recovery to decreasing sulfur depositions and to more favorable weather conditions.

### Deposition

Both bulk and throughfall deposition of $S\text{-}SO_4^{2-}$ show high variations among the Level II plots and marked spatial patterns (Fig. 2). Shares of plots with the highest deposition ranging from 8.0–27.1 kg • ha$^{-1}$ • year$^{-1}$ are particularly frequent in Belgium, the Czech Republic, the Slovak Republic, and Northern Italy. In Belgium, high sulfate deposition coincides with high sodium deposition, indicating sea salt as an origin. In the other regions, the high deposition is likely to reflect regional emission situations. Bulk and throughfall deposition of $S\text{-}SO_4^{2-}$ is particularly low in Norway, Sweden, Finland, and Latvia. Throughfall deposition is clearly higher than bulk deposition, indicating dry deposition filtered from the air by the canopy and washed off the leaves.

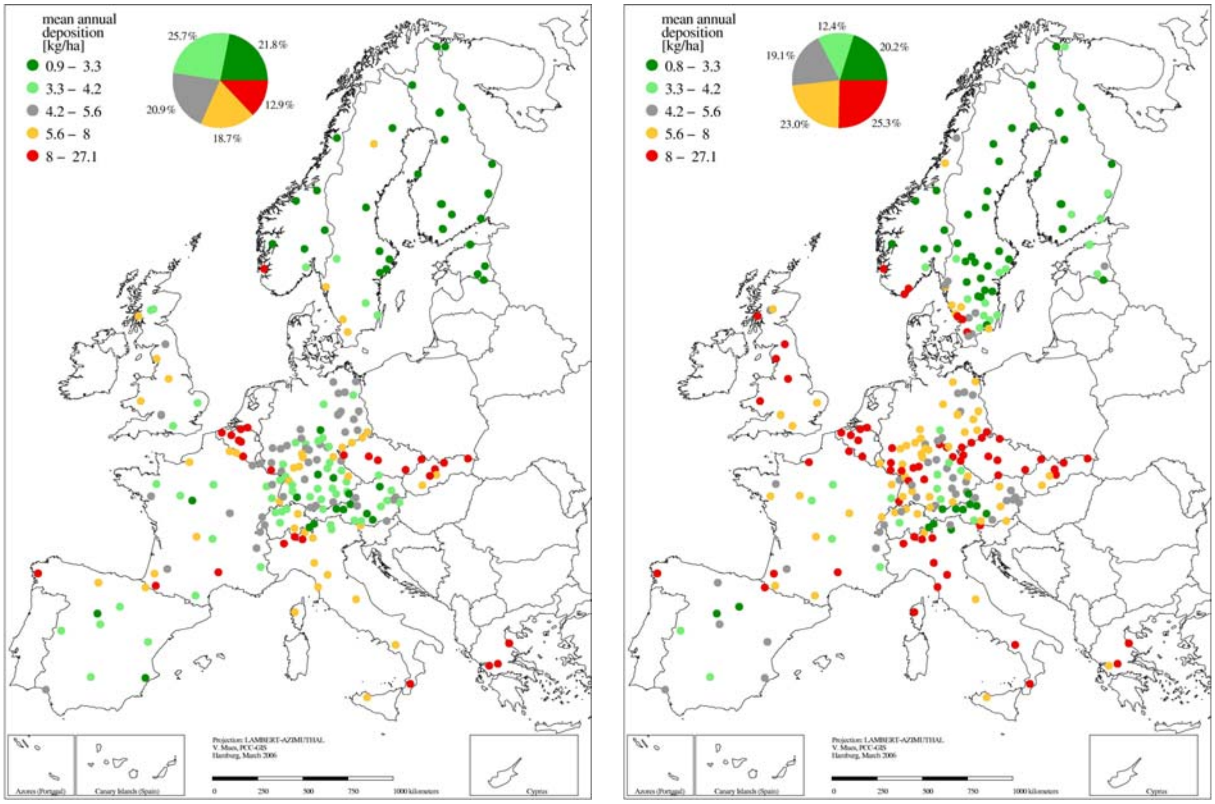

**FIGURE 2.** Mean annual bulk (left) and throughfall (right) deposition of $S\text{-}SO_4^{2-}$ from 2001–2003.

The changes in bulk and throughfall deposition of $S\text{-}SO_4^{2-}$ from 1998–2003 are shown in Fig. 3. In the pie diagrams, the shares of plots showing significant decreases (error probability lower or equal 5%) and decreases (error probability larger than 5%) amount to 85.6% for bulk deposition and to 90.6% for throughfall deposition. This indicates a general decrease in the deposition of $S\text{-}SO_4^{2-}$ within the observation period. Plots with decreasing deposition are scattered across Europe without showing any spatial pattern.

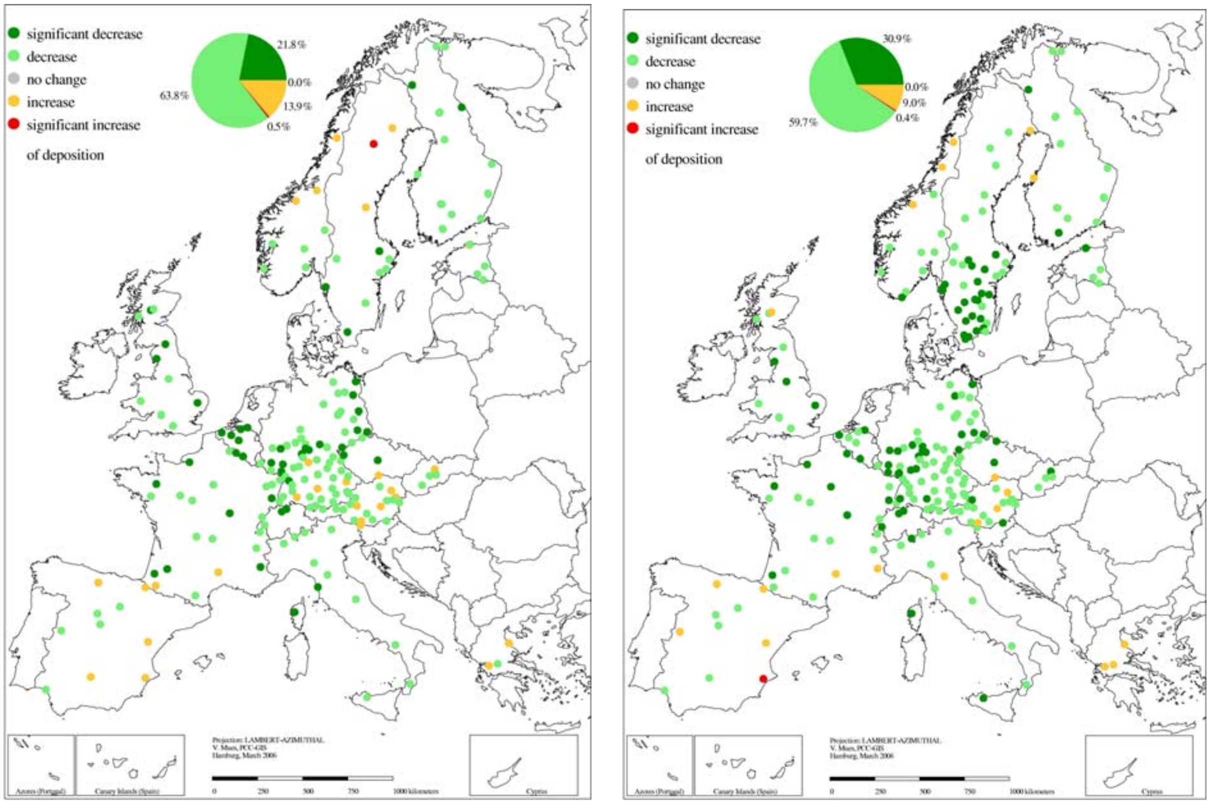

**FIGURE 3.** Temporal change in bulk (left) and throughfall (right) deposition of $S\text{-}SO_4^{2-}$ from 1998–2003.

In Fig. 4, the changes of bulk and throughfall deposition of $S\text{-}SO_4^{2-}$, $N\text{-}NH_4^+$, and $N\text{-}NO_3^-$ from 1998–2003 are compared. It is shown that bulk deposition of $S\text{-}SO_4^{2-}$ decreases from 6.5–4.3 kg • ha$^{-1}$ • year$^{-1}$. Throughfall deposition of $S\text{-}SO_4^{2-}$ is larger than bulk deposition and decreases from 9.3–5.8 kg • ha$^{-1}$ • year$^{-1}$. The fact that the decrease in throughfall deposition is more pronounced than that in bulk deposition may be explained by the dry deposition washed off the leaves. Compared to $S\text{-}SO_4^{2-}$, bulk and throughfall deposition of $N\text{-}NH_4^+$ and $N\text{-}NO_3^-$ are lower. Moreover, bulk deposition of the two nitrogen compounds decreases far less and the throughfall deposition shows hardly any trend.

Decreasing bulk and throughfall deposition may well reflect a decrease in precipitation. In fact, precipitation was found to decrease in the period of observation and the dry summer in 2003 seems to be reflected in Fig. 4 by particularly low depositions in that year. However, the decrease in sulfate depositions by about one-third and the different development of nitrogen depositions can hardly be explained by decreasing precipitation. A previous study showed that sulfate concentrations in precipitations are markedly reduced, whereas nitrogen concentrations hardly decreased[10]. This reflects the reduction of sulfur emissions under CLRTAP politics over the last years[11] and the less-pronounced reduction of nitrogen emissions in Europe. This interpretation is also supported by findings[3,4] showing decreasing sulfate depositions and a recuperation of forest soils from acidification. These findings and the results of the present study indicate that air pollution control under CLRTAP shows the first positive effects on forests in Europe. The results of the deposition measurements alone do not permit drawing conclusions on air pollution effects on forests. Besides the levels of atmospheric concentrations and depositions of air pollutants, a multitude of further site conditions, such as soil type, tree species, stand age, and stand structure, are decisive for the occurrence of potential harmful effects at a given site. Therefore, reliable risk assessments require calculations of critical loads and levels as well as their exceedances for each pollutant on each individual plot. The data presented here are indispensable for such studies.

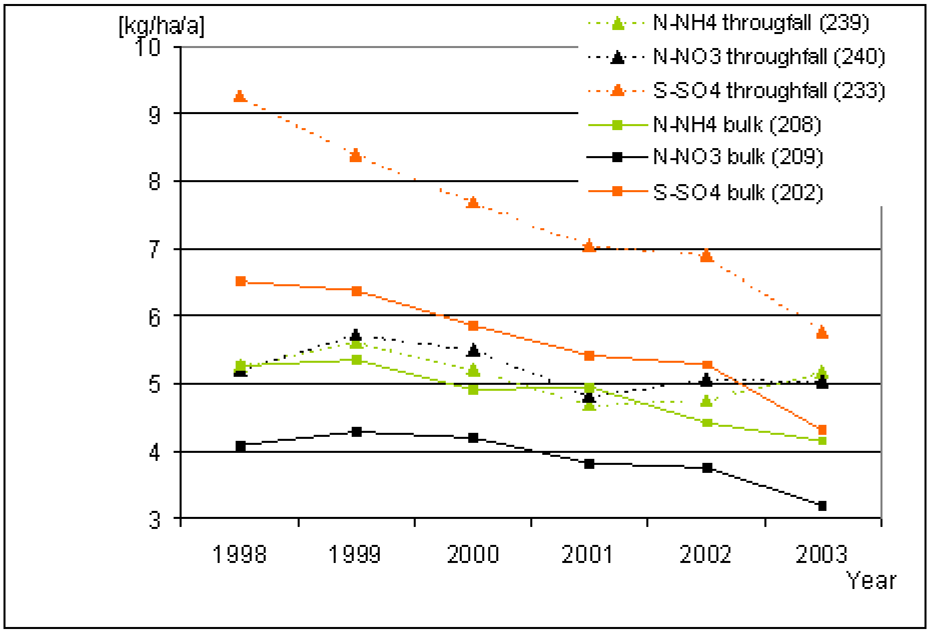

**FIGURE 4.** Temporal changes in bulk and throughfall deposition of S-$SO_4^{2-}$, N-$NH_4^+$, and N-$NO_3^-$ from 1998–2003.
